# The auxin signaling pathway contributes to phosphorus-mediated zinc homeostasis in maize

**DOI:** 10.1186/s12870-023-04039-8

**Published:** 2023-01-10

**Authors:** Zhonghua Wang, Yafei Wang, Qingguo Du, Pengshuai Yan, Baogang Yu, Wen-Xue Li, Chun-Qin Zou

**Affiliations:** 1 College of Resources and Environmental Sciences; National Academy of Agriculture Green Development; Key Laboratory of Plant-Soil Interactions, Ministry of Education, China Agricultural University, Beijing, 100193 People’s Republic of China; 2 National Engineering Laboratory for Crop Molecular Breeding, Institute of Crop Science, Chinese Academy of Agricultural Sciences, Beijing, 100081 China

**Keywords:** Auxin, RSA, miR167, P-mediated Zn homeostasis, Zn localization, Maize

## Abstract

Although the interaction between P and Zn has long been recognized in plants, the physiological and molecular mechanisms underlying P and Zn interactions are poorly understood. We show here that P supply decreases the Zn concentration in maize shoots and roots. Compared to +P + Zn (addition of both P and Zn), +P-Zn reduced and -P-Zn increased the total length of 1° lateral roots (LRs). Under +P + Zn, both P and Zn concentrations were lower in the *sl1* mutant roots than in wild-type (WT) maize roots, and P accumulation did not reduce the Zn concentration in *ll1* mutant roots. Transcriptome profiling showed that the auxin signaling pathway contributed to P-mediated Zn homeostasis in maize. Auxin production and distribution were altered by changes in P and Zn supply. Cytosolic Zn co-localized with auxin accumulation under +P + Zn. Exogenous application of 1-NAA and L-Kyn altered the P-mediated root system architecture (RSA) under Zn deficiency. -P-Zn repressed the expression of miR167. Overexpression of *ZmMIR167b* increased the lengths of 1° LRs and the concentrations of P and Zn in maize. These results indicate that auxin-dependent RSA is important for P-mediated Zn homeostasis in maize.

**Highlight**

Auxin-dependent RSA is important for P-mediated Zn homeostasis in maize.

**Supplementary Information:**

The online version contains supplementary material available at 10.1186/s12870-023-04039-8.

## Background

As sessile organisms, plants must often cope with insufficient availability of multiple nutrients in soil. Normal plant growth requires at least 16 nutrients, including phosphorus (P) and zinc (Zn). Zn is the only mineral nutrient that functions as a component and/or structural co-factor of all six major groups of plant enzymes [1]. Zn deficiency is estimated to affect about one-third of the world’s human population, and especially those who live on plant-based diets in developing countries [2–4]. P is a key environmental factor limiting crop production, and much more attention has been paid to P-use efficiency than to Zn-use efficiency of crops [5, 6].

Inorganic phosphorus (Pi) can affect the bioavailability and mobility of metal elements such as Zn in soil [7]. The homeostasis of P and Zn is interconnected in plants. Deficiency or excess in one element affects the concentration of the other [8, 9]. The following four main types of interactions between P and Zn in plants have long been recognized: (1) P accumulation induced by Zn deficiency [7, 10]; (2) Zn accumulation induced by P deficiency [11]; (3) P deficiency induced by a high level of Zn [12, 13]; and (4) Zn deficiency induced by P application [14, 15]. Unfortunately, all four types of interactions between P and Zn homeostasis will reduce crop yield and quality. Strategies that balance P and Zn levels in plants are therefore needed to optimize crop yield and quality. Obtaining that balance will require a detailed understanding of the physiological and molecular mechanisms linking P/Zn interactions.

Progress in understanding the molecular interactions between P and Zn has recently been made, especially with regard to P accumulation induced by Zn deficiency. In barley, Zn deficiency upregulated the expression levels of two high-affinity Pi transporters, *HvPHT1* and *HvPHT2*, under both P-sufficient and -deficient conditions, resulting in an over-accumulation of P in shoots [16]. In *Arabidopsis thaliana*, in contrast, Zn deficiency induced the expression of a high-affinity Pi transporter gene, *AtPHT1;1*, in shoots with concurrent down-regulation in roots [17]. The mRNA abundance of *AtPHO1;H3* was enhanced by Zn deficiency, and the upregulation of *AtPHO1;H3* could limit P transfer from roots to shoots [8]. Under Zn-deficient conditions, *LPCAT1* encodes a lysophosphatidylcholine acyltransferase and controls P accumulation in *Arabidopsis* shoots by modulating phospholipid metabolism and Pi transporter expression [9].

As noted in the first paragraph, Zn deficiency is a serious problem among humans, a problem that is made worse by P fertilization. Farmers have often applied excessive quantities of P fertilizer to obtain high yields [18]. These large inputs of external P with decreasing P-use efficiency result in P accumulation in soil and Zn deficiency in plants [14, 15] and therefore in the human diet. Research is needed to determine the molecular basis of how P accumulation in soil and plants results in Zn deficiency in plants.

Plants must alter the development of individual roots to form an optimized root system architecture (RSA) for exploration and uptake of mineral nutrients in soil [19]. Although researchers traditionally thought that each individual nutrient deficiency induced a typical architecture [20], evidence increasingly indicates that plants integrate multiple nutritional stimuli into complex developmental programs that control RSA [19, 21]. Pi deficiency generally inhibits primary root growth, stimulates axial branching, and produces a horizontal growth angle of adventitious roots [22, 23]. Compared to Pi deficiency, Zn deficiency has an opposite effect on RSA, and promotes primary root growth. However, these observations were mainly with the model plant *Arabidopsis thaliana*. Similar responses including Pi deficiency-mediated inhibition of primary root growth were not observed in rice or maize [24]. We do not know whether Pi and Zn deficiency together would have additive effects on RSA or would result in a new RSA in maize.

P regulates RSA by the local perception of PO_4_^−^ at the root tip, which affects the levels of multiple plant hormones, including auxin, strigolactones, cytokinins, gibberellins, and ethylene [25]. Gao et al. reported that a cytokinin-dependent regulatory module underlies the maintenance of Zn nutrition in rice [26]. These results indicate that changes in levels of plant hormones might underlie the altering RSA under Pi deficiency and Zn starvation conditions. Here, we show that lateral root (LR) traits in maize are affected by P and Zn supplies. By comparing transcriptome profiling of LR treated or not treated with P and Zn, determining auxin content and distribution, rescuing by the exogenous application of NAA and L-Kyn, and constructing LR mutants and miR167 transgenic maize, we demonstrate that auxin is involved in the interactions between P and Zn that determine RSA in maize.

## Results

### Exogenous P decreases Zn concentration in maize

**Fig. 1:**
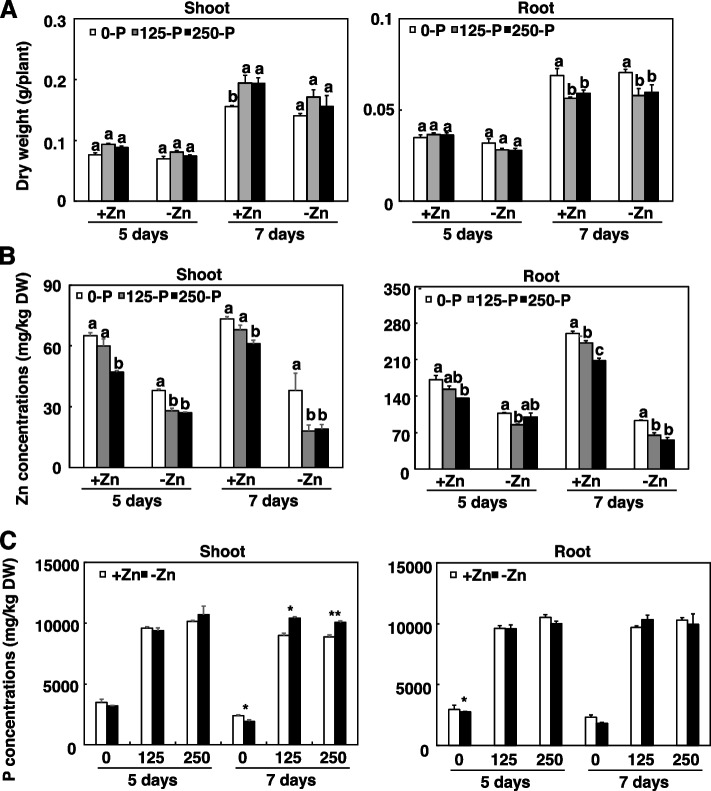
P and Zn concentrations in shoots and roots of maize supplied with different combinations of P and Zn. Inbred line B73 plants were grown in a hydroponic solution containing different combinations of P and Zn at the indicated durations before shoot and root dry weights (**A**) and concentrations of Zn (**B**) and P (**C**) in shoots and roots were determined. Values are means ± SD (*n* = 4). Means with the same letter are not significantly different at *P* < 0.05 according to the LSD test. *P < 0.05 and **P < 0.01 (Student’s *t*-test) indicates a significant difference from the control.

To investigate the interaction between P and Zn, we grew maize inbred line B73 under one of the six combinations of P and Zn supply. Short-duration (5 days) P and Zn treatments did not significantly affect the shoot or root dry weight of maize (Fig. 1A). When P and Zn treatments were extended to 7 days, +P increased shoot dry weight when combined with +Zn but reduced root dry weight when combined with +Zn (Fig. 1A). At 7 days, +P significantly decreased the Zn concentrations in maize roots and shoots (Fig. 1B). For solutions with +Zn, decreases in the Zn concentrations in maize roots and shoots were negatively related to the quantity of P supplied (Fig. 1B). In contrast, P accumulation induced by Zn deficiency was observed only in maize shoots after long-duration (7 days) P and Zn treatments (Fig. 1C). These results suggested that Zn deficiency induced by P application precedes P accumulation induced by Zn deficiency in maize. We therefore focused on P-mediated Zn homeostasis in the following experiments.

### Effects of P availability and Zn deficiency on the RSA of maize

**Fig. 2:**
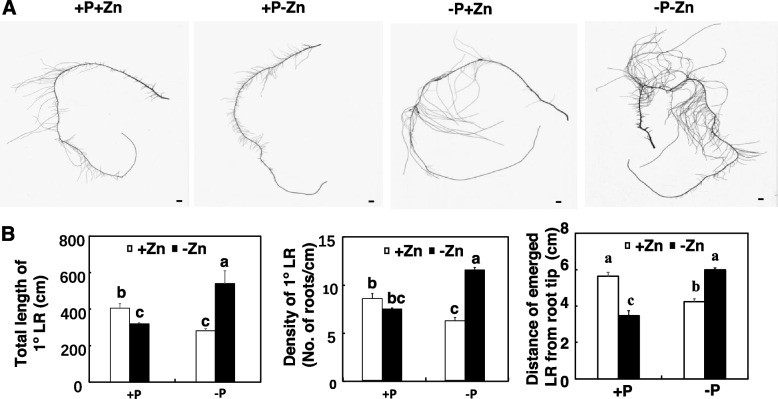
Effects of P and Zn supply on root system architecture (RSA) of primary roots of maize. Inbred line B73 was grown in a hydroponic solution containing different combinations of P and Zn supply for 7 days. **A** The representative images of primary roots of maize taken with a flatbed scanner. Scale bars = 1 cm. **B** Effects of P and Zn supply on total root length, density of 1° lateral roots (LRs), and distance of emerged LRs from the root tip. Values are means ± SD (*n* = 12). Means with the same letter are not significantly different at *P* < 0.05 according to the LSD test

When challenged by P and Zn deficiency, plants optimize their RSA to maximize the exploration and uptake of P and Zn [3, 27]. We first investigated the effects of P availability on the RSA of maize during Zn deficiency. Compared with +P+Zn, +P-Zn and -P+Zn, -P-Zn significantly increased the total root length about 39, 47 and 32%, respectively (Fig. S1). Because of the large size of maize roots, we used the primary root and its lateral roots (LRs) as representative of the whole root system for analyzing the effects of P availability on the RSA of maize during Zn deficiency. Consistent with the observations of whole root systems, the total length of the primary root (primary root and LRs) was longer with -P-Zn than with +P+Zn, +P-Zn or -P+Zn (Fig. S2). P availability did not affect primary root length during Zn deficiency (Fig. S2). However, the total root length of 1° LRs and density of 1° LRs were significantly higher under -P-Zn than under +P+Zn, -P+Zn or + P-Zn (Fig. 2A and B). Relative to +P+Zn, +P-Zn inhibited the total root length of 1° LRs and reduced the distance between the site where the 1° LR emerged and the root tip (Fig. 2A and B). These results indicated that LR traits are affected by P and Zn deficiency.

### LR mutants have an altered Zn homeostasis

**Fig. 3:**
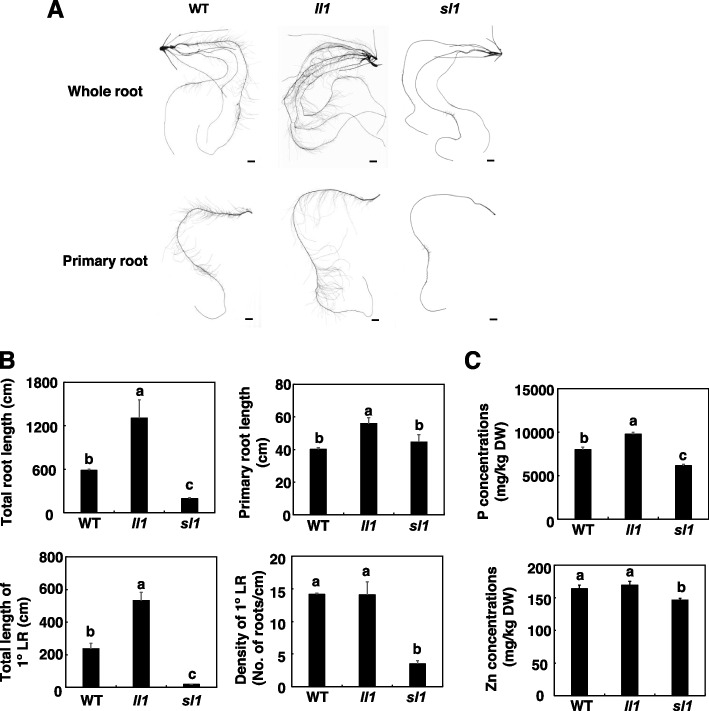
P and Zn concentrations in lateral root (LR) mutants of maize. LR mutants *ll1* and *sl1* were grown in a hydroponic solution containing different combinations of P and Zn for 7 days. **A** Representative images of whole root systems and primary roots of *ll1* and *sl1*. Scale bars = 1 cm. **B** Total root length, primary root length, total root length of 1° LRs, and density of 1° LRs of *ll1* and *sl1*. Values are means ± SD (*n* = 12). **C** P and Zn concentrations in the roots of *ll1* and *sl1* mutants. Values are means ± SD (*n* = 4). Means with the same letter are not significantly different at *P* < 0.05 according to the LSD test

To further characterize the function of LRs in P-mediated Zn homeostasis in maize, we searched our collected EMS mutant lines and found two genetically stable LR mutants. One mutant had a long primary root and LRs, and was designated *ll1* (http://elabcaas.cn/memd/public/index.html#/, mutant ID: EMS4-16d24b) [28]. *ll1* contains a G/A substitution at nucleotide 897 after the ATG codon of *Zm00001d007971* (unknown protein), which leads to a premature stop codon in the gene. The total length of the whole root and primary roots in *ll1* was 1306 cm and 532 cm, respectively, which were about 2.3 and 2.2 times longer than in the wild type (WT) (Fig. 3A and B). In contrast, another mutant had short and sparse LRs, and was designated *sl1* (Fig. 3A and B). *sl1* is also a stop-gained mutant and contains a G/A substitution at nucleotide 789 after the ATG codon of *Zm00001d045571* (unknown protein).

We grew the two LR mutants in hydroponic solutions containing combinations of P and Zn. Under +P+Zn, both P and Zn concentrations were lower in the *sl1* mutant than in the WT (Fig. 3C). Root P concentrations in the *ll1* mutant were 22% higher than in the WT. Interestingly, P accumulation did not reduce the Zn concentration in roots of the *ll1* mutant (Fig. 3C). These results suggested that LRs are important in P-mediated Zn homeostasis in maize.

### Transcriptome profiling of LRs treated with combinations of P and Zn supply

**Fig. 4:**
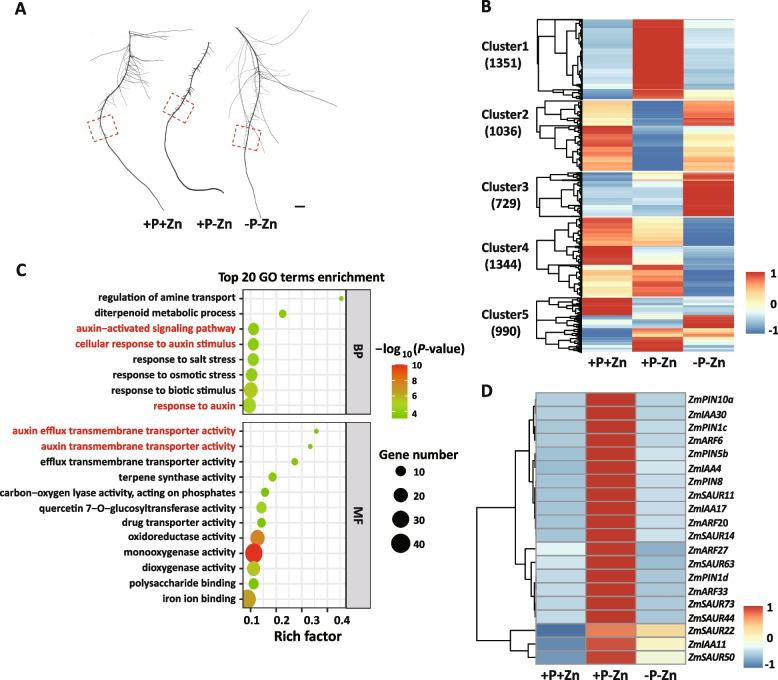
Effects of P and Zn supply on transcriptome profiling of lateral roots emerging from the primary roots of maize. Inbred line B73 were grown in a hydroponic solution containing different combinations of P and Zn for 7 days. **A** Diagram of sampling position. The red-dotted box indicates the sampling location on the primary root for RNA-seq. Scale bars = 1 cm. **B** Expression pattern clustering of differentially expressed genes (DEGs) in response to +P-Zn and -P-Zn compared with +P+Zn. Cluster 1: specifically induced by +P-Zn; Cluster 2: specifically repressed by +P-Zn; Cluster 3: specifically induced by -P-Zn; Cluster 4: specifically repressed by -P-Zn (cluster 4, 1344 genes); Cluster 5: different response to +P-Zn and -P-Zn than to +P + Zn. The gradient color scale indicates gene expression levels, which were normalized by the Z-score of gene expression across the three samples. **C** Gene ontology (GO) enrichment of DEGs in cluster 1. The scatter plot displays the 20 highest enriched terms. The enrichment factor is the ratio of DEG numbers in the GO entry term to all gene numbers in this pathway term. The point size represents the number of genes in the pathway; the point color meant –log_10_(*P*-value). **D** List of genes involved in the auxin signaling pathway in cluster 1. The gradient color scale indicates gene expression levels, which were normalized by the Z-score of gene expression across three samples

To gain insight into the molecular events involved in P-mediated Zn homeostasis in maize, we compared the transcriptome profiling of maize root under +P+Zn, +P-Zn, and -P-Zn. Total RNA was extracted from the root at the site where LRs began to emerge (Fig. 4A). Each sample was represented by three biological replicates. The nine RNA libraries yielded more than 0.28 billion raw reads, and only those that were perfectly mapped to maize B73 RefGen_V4 (ftp://ftp.ensemblgenomes.org/pub/plants/release-41/fasta/zea_mays/dna/) were analyzed further. The abundance of each gene was expressed as fragments per kilo base million mapped reads (FPKM) [29]. The Pearson’s correlation coefficients of the three biological replicates exceeded 0.99, indicating a high correlation between biological replicates (Fig. S3).

With a threshold fold-change of ≥2 and an adjusted P ≤ 0.05, 3377 and 3063 differentially expressed genes (DEGs) relative to +P+Zn were identified under +P-Zn and -P-Zn, respectively. The results of RNA-seq were confirmed by quantitative real-time RT-PCR. In agreement with our RNA-seq data, the expression levels of randomly selected Zm00001d032850 and Zm00001d038437 were expressed at higher levels under +P-Zn and -P-Zn than +P+Zn (Fig. S4). As expected, the expression level of Zm00001d027700 was repressed by +P-Zn and induced by -P-Zn (Fig. S4), demonstrating the reliability of our RNA-seq data. We further divided these DEGs into five groups: (1) specifically induced by +P-Zn (cluster 1, 1351 genes); (2) specifically repressed by +P-Zn (cluster 2, 1036 genes); (3) specifically induced by -P-Zn (cluster 3, 729 genes); (4) specifically repressed by -P-Zn (cluster 4, 1344 genes); and (5) different response to +P-Zn and -P-Zn than to +P + Zn (cluster 5, 990 genes) (Fig. 4B). GO analysis (http://systemsbiology.cau.edu.cn/agriGOv2/) indicated that the DEGs in the five groups have known or presumed functions associated with abiotic stress responses. Interestingly, the annotated DEGs in cluster 1 were enriched in auxin efflux transmembrane transporter activity (GO:0010329, P = 0.00034), auxin-activated signaling pathway (GO:0009734, P = 0.00017), and response to auxin (GO:0009733, P = 2.70e-5) (Fig. 4C), including AUXIN RESPONSE FACTOR (ARF), AUX/IAA transcription factor, small auxin up-regulated RNA (SAUR), and PIN-FORMED auxin efflux transporters (PINs) (Fig. 4D). These results indicated that auxin signaling pathway might contribute to P-mediated Zn homeostasis in maize.

### Effects of P availability on auxin content and distribution in LRs of maize subjected to Zn deficiency

**Fig. 5:**
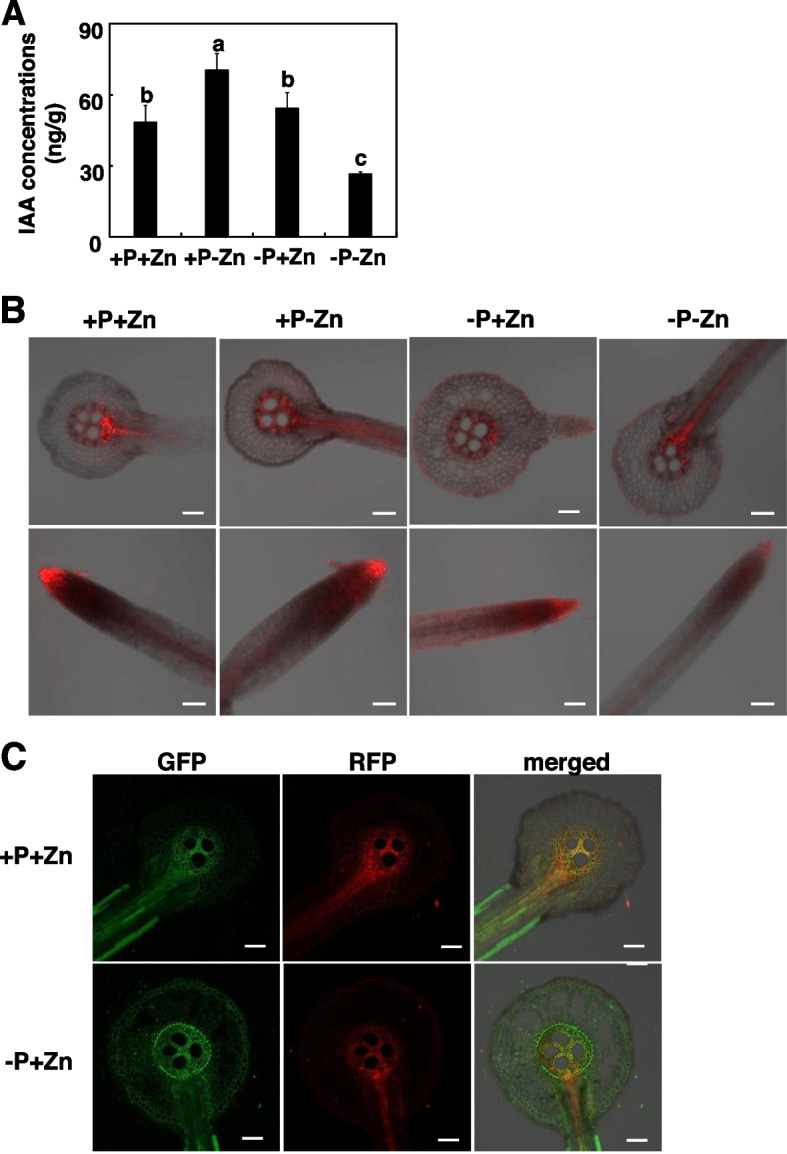
Effects of P and Zn supply on the production and distribution of auxin in maize. Inbred line B73 were grown in a hydroponic solution containing different combinations of P and Zn for 7 days. **A** Effects of P and Zn supply on free indole-3-acetic acid in lateral roots emerging from the primary root. Values are means ± SD (*n* = 4). Means with the same letter are not significantly different at *P* < 0.05 according to the LSD test. **B** Effects of P and Zn supply on fluorescence of *ZmDR5::mRFP* reporter maize. Scale bars = 100 μm. **C** The distribution of cytosolic Zn in roots of *ZmDR5::RFP* reporter maize. Cytosolic Zn was visualized by the membrane-permeant fluorescent sensor Zinpyr-1. Scale bars = 100 μm

To test the hypothesis that auxin might contribute to P-mediated Zn homeostasis in maize, we first determined the free IAA content in roots at the site where LRs began to emerge (Fig. 4A). The free IAA content was 70.47 ng g^− 1^ and 54.04 ng g^− 1^ FW (fresh weight) in emerging LRs under +P-Zn and, which was 46 and 12% higher than under +P+Zn, respectively (Fig. 5A). In contrast, -P-Zn significantly reduced the free IAA content in emerging LRs, and the free IAA content was ~ 50% lower under -P-Zn than under +P+Zn (Fig. 5A). These results suggested that P addition affected auxin production in LRs subjected to Zn deficiency.

Transcriptome profiling revealed that PINs responded differently to +P-Zn vs. -P-Zn. This indicated that P availability might affect auxin distribution during Zn deficiency. To test this hypothesis, we used the auxin-responsive *ZmDR5::RFP* reporter maize to examine local auxin accumulation under +P+Zn, +P-Zn, -P+Zn and -P-Zn. Consistent with the free IAA content, the RFP signals were reduced in LR caps under -P-Zn (Fig. 5B). Longitudinal sections through LRs showed that auxin accumulation was highest at the vascular connection with the parent root under +P+Zn and -P-Zn. In contrast, the RFP signals were dispersed throughout the endodermis under +P-Zn (Fig. 5B).

We also used the membrane-permeant fluorescent sensor Zinpyr-1 to image cytosolic Zn. In inbred line B73 under +P+Zn, the fluorescence was mainly located at the vascular connection with the parent root (Fig. S5), which is where auxin accumulation was highest. We therefore used *ZmDR5::RFP* reporter maize to determine whether cytosolic Zn co-localized with auxin in LRs. Under +P+Zn but not under -P+Zn, Zinpyr-1 fluorescence co-localized with RFP signals at the vascular connection with the parent root (Fig. 5C). These results indicated that P addition affected auxin production and distribution in LRs subjected to Zn deficiency.

### NAA and L-Kyn alter P-mediated RSA under Zn deficiency

**Fig. 6:**
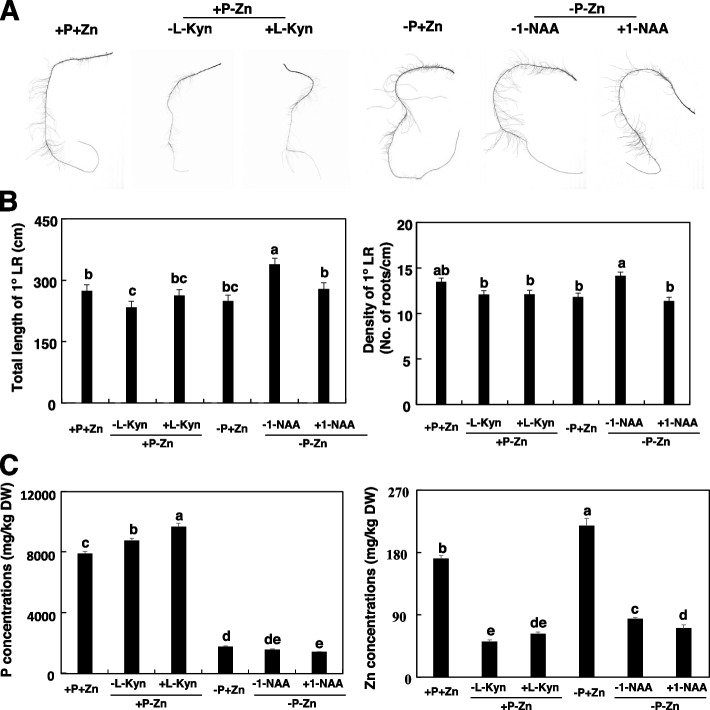
Effects of L-Kyn and 1-NAA on root system architecture (RSA) of primary maize roots under +P-Zn and -P-Zn. Inbred line B73 were grown in a hydroponic solution containing different combinations of P and Zn for 7 days. **A** Representative images of the primary roots of inbred line B73 treated with L-Kyn and 1-NAA under +P-Zn and -P-Zn. Scale bars = 1 cm. **B** Effects of L-Kyn and 1-NAA on the total length and density of 1° lateral roots of the primary root under +P-Zn and -P-Zn. Values are means ± SD (*n* = 12). **C** P and Zn concentrations in the roots. Values are means ± SD (*n* = 4). Means with the same letter are not significantly different at *P* < 0.05 according to the LSD test

To further verify that P-mediated RSA under Zn deficiency is caused by auxin homeostasis, we added the auxin analog 1-naphthaleneacetic acid (1-NAA) and the auxin synthesis inhibitor L-Kyn to hydroponic solutions containing different combination of P and Zn [30]. Maize subjected to +P-Zn and -P-Zn was treated with 20 μM L-Kyn or 20 mM 1-NAA for 7 days. L-Kyn increased the total length of 1° LRs under +P-Zn. The total length of 1° LRs was 269.2 cm under +P-Zn + L**-**Kyn, which was similar to that under +P+Zn (Fig. 6A and B). In contrast, application of 1-NAA significantly reduced the total length and density of 1° LRs under -P-Zn (Fig. 6A and B). In agreement with the phenotypes of 1° LRs, the P concentrations in maize roots were higher under +P-Zn + L**-**Kyn than under +P-Zn-L**-**Kyn (Fig. 6C). In contrast, application of 1-NAA significantly reduced the total length and density of 1° LRs and Zn concentrations in maize roots under -P-Zn (Fig. 6A and B). These results further suggested that auxin homeostasis is involved in P-mediated RSA under Zn deficiency.

### miR167 is involved in P-mediated Zn homeostasis in maize

**Fig. 7:**
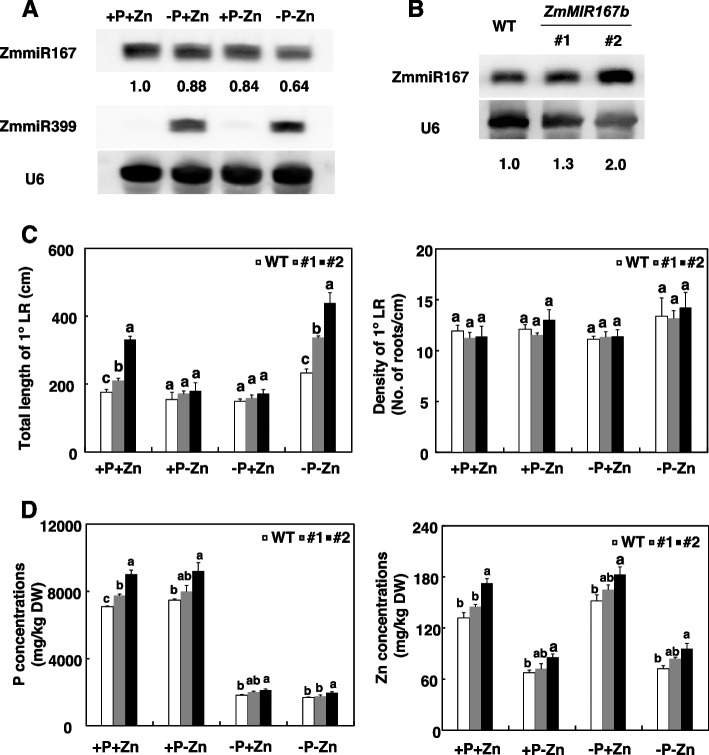
ZmmiR167 is involved in P-mediated Zn homeostasis in maize. Inbred line B73 were grown in a hydroponic solution containing different combinations of P and Zn for 7 days. **A** Regulation of ZmmiR167 by P and Zn supply. miR399 or U6 RNA was probed as a loading control. **B** Overexpression of ZmmiR167b in transgenic maize. RNA gel blot analysis of ZmmiR167 levels in the wild type (WT) and two representative transgenic lines. U6 RNA was used as a loading control. Numbers below each lane indicate relative expression. **C** Total root length and density of 1° LRs of WT and ZmMIR167b transgenic maize as affected by P and Zn supply. Values are means ± SD (*n* = 12). **D** P and Zn concentrations of WT and ZmMIR167b transgenic maize as affected by P and Zn supply. Values are means ± SD (*n* = 4). Means with the same letter are not significantly different at *P* < 0.05 according to the LSD test

ARFs determine plant response to auxin and are subtly regulated by miR160, miR167, and miR390 [31–33]. The DEGs in out transcriptome profiling included ARFs and miR167s, indicating that miR167 might affect P-mediated Zn homeostasis in maize. To test this hypothesis, we first investigated miR167 responses to different P and Zn supplies. The expression levels of miR167 were lower under -P+Zn, +P-Zn, and -P-Zn than under +P+Zn (Fig. 7A; Fig. S6). miR167 abundance was 36% lower under -P-Zn than under +P+Zn (Fig. 7A). We therefore used *ZmMIR167b* overexpressing transgenic maize (line #1 and #2) to determine the roles of miR167 in P-mediated Zn homeostasis in maize (Fig. 7B; Fig. S7). Overexpression of *ZmMIR167b* did not affect the density of 1° LRs of maize under different P and Zn supplies (Fig. 7C; Fig. S8). However, the total length of 1° LRs was significantly greater in *ZmMIR167b* overexpressing transgenic maize than in the WT under +P + Zn and -P-Zn (Fig. 7C). Both P and Zn concentrations were higher in *ZmMIR167b* overexpressing transgenic maize than in WT maize, especially in line #2 (Fig. 7D).

## Discussion

**Fig. 8:**
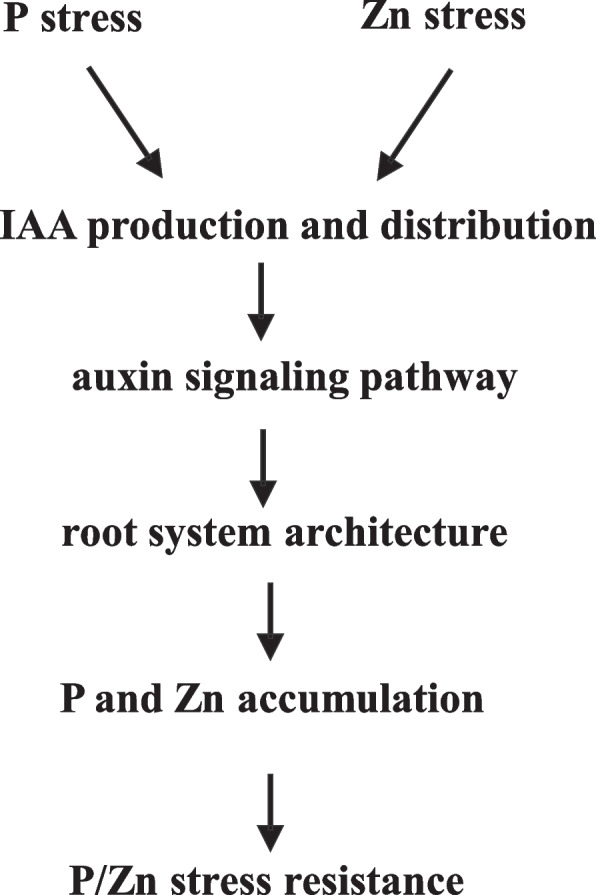
A proposed model of phosphorus-mediated zinc homeostasis in maize

P application immobilizes Zn in soil and therefore reduces Zn uptake by plants [11, 34]. P application also increases plant biomass and can therefore reduce the Zn concentration in plants not only by reducing Zn uptake but also by biomass dilution [35]. In the current study, P addition reduced root dry weight, increased P concentrations, and decreased Zn concentrations in maize. These results indicated that factors other than biomass could affect P and Zn homeostasis in maize. P is more available in the upper soil layers, and a shallower RSA was found to favor topsoil foraging for P [23]. Our previous research also showed that Zn accumulation in wheat shoots was positively correlated with the concentration of available Zn in soil, especially in the 0-30 cm soil layer, and that a shallow RSA was also required for Zn uptake [3, 36]. These results indicated that RSA changes induced by nutrient supply could help explain the interactions between P and Zn in plants. In the present research, we demonstrated that P and/or Zn stresses affected auxin production and distribution in roots, which could activate the expression of genes involved in auxin signaling pathway. As a consequence, the RSA changed and contributed to P-mediated Zn homeostasis in maize (Fig. 8). To our knowledge, this is the first report to describe the involvement of auxin in the interaction between P and Zn in plants.

Auxin is a key player in RSA changes induced by nutrient deficiency [7, 37, 38]. Auxin is associated with the Pi-starvation signaling that affects LR development at multiple steps preceding LR formation [39]. In contrast, the functions of auxin in plant adaptation to Zn stress have seldom been considered. In the current research with maize, we found that (1) cytosolic Zn co-localized with auxin in LRs under +P+Zn; (2) +P-Zn significantly enhanced the free IAA content in LRs emerging from primary roots; (3) +P-Zn altered local auxin accumulation in LRs; (4) L-Kyn increased the total length of 1° LRs under +P-Zn; (5) DEGs in +P-Zn and -P-Zn compared with +P+Zn were enriched in the auxin signaling pathway; and (6) overexpression of miR167 affected P and Zn homeostasis. These results indicated that auxin plays important roles in the adaptation of maize to Zn stress.

In *Arabidopsis*, induction of LRs is an important mechanism for adapting to deficiencies in P, K, Zn, and Mn [40]. However, research has increasingly indicated that induction of LRs in response to Pi-deficient conditions may not be a common response in other plant species [39]. Here, we found that +P-Zn significantly reduced the total length but not the density of 1° LRs in maize. Consistent with the effects of +P-Zn, application of the auxin homologue 1-NAA and the auxin synthesis inhibitor L-Kyn mainly affected the total length of 1° LRs. In addition, the total length but not density of 1° LRs altered in *ll1* mutant/miR167 overexpressing transgenic maize compared with WT maize. We therefore concluded that the total length but not the density of 1° LRs is important in P and Zn homeostasis in maize.

The interaction between P and Zn in crops always reduces their yield and quality. In the current study, for example, P accumulation induced Zn deficiency in plants. In the maize *ll1* mutant (long LRs and primary roots), we found that P accumulation did not reduce root the Zn concentration under +P+Zn. Overexpression of miR167 enhanced the total length of 1° LRs and P and Zn concentrations under +P+Zn. These results indicated that maize plants could overcome the adverse effects of P accumulation by increasing the total length of their 1° LRs.

## Materials and methods

### Plant materials and growth conditions

*ZmMIR167b* overexpressing transgenic maize in the Chang 7-2 genetic background was kindly provided by Zhongfu Ni (China Agricultural University). *ZmDR5::RFP* reporter maize in the B73 genetic background were kindly provided by Fang Yang (Huazhong Agricultural University)*.* The LR mutants *ll1* and *sl1* in the B73 genetic background were collected in our laboratory. Seed-surface sterilization and germination were performed as previously described [41]. After their endosperms were removed, the seedlings were transferred to 3-L containers containing Hoagland’s solution with or without additional P (KH_2_PO_4_, 0.25 mmol/L) or additional Zn (ZnSO_4_·7H_2_O, 1 μmol/L). This resulted in six treatment combinations, i.e., 250P+Zn (+P+Zn), 125+Zn, 0P+Zn (-P + Zn), 250P-Zn (+P-Zn), 125P-Zn, 0P-Zn (-P-Zn), in which the plus or minus sign indicates that the P or Zn was or was not included. The maize plants were grown hydroponically in a growth chamber at 28:22 °C day:night with 14 h:10 h light:dark. The samples were collected at the indicated times after initiation of P and Zn treatments.

### RNA analysis

Total RNA was extracted from maize and *Arabidopsis* by TransZol Up (TransGen Biotech, China). Real-time RT-PCR and enrichment, fractionation, and detection of miRNA were performed as previously described [42]. In briefly, the first-strand cDNA was synthesized using SuperScript III first-strand synthesis supermix (Invitrogen). The expression levels of *Zm00001d032850*, *Zm00001d027700* and *Zm00001d038437* were detected with specific primers. qPCR was carried out in an ABI 7500 system (Applied Biosystems) using the SYBR PreMix Ex Taq (Perfect Real Time) kit (Takara Biomedals). Each experiment was replicated three times. The sequences of the specific primers are listed in Table S1.

### RNA-seq analysis

Total RNA was extracted from primary roots where a lateral root (LR) began to emerge. After digestion with DNase I (TaKaRa, Japan), RNA was purified, and mRNA was enriched. RNA-sequencing libraries were constructed with the NEBNext Ultra Directional RNA Library Prep Kit for Illumina (NEB, USA), and the libraries were sequenced with the Illumina Hiseq 2500 platform (Berry Genomics, Beijing, China).

Clean data were obtained by excluding low-quality reads and adapter sequences using fastp software [43]. The unique reads were aligned to B73_RefGen_v4.41 using HISAT2 v2.1.0 with default parameters [44]. The count information was calculated with featureCounts software [45]. Differentially expressed genes (DEGs) were identified with the edgeR package [46]. Genes were considered to be differentially expressed between two treatments if the log_2_ fold-change ratio was ≥1 and if the adjusted *P* value was < 0.05. Gene ontology (GO) enrichment analyses were performed using AgriGO v2.0 with Maize AGPv4 as the reference background [47].

### Analysis of RSA

The roots were placed in a transparent tray. Root images from at least 10 roots per treatment were obtained using a flatbed scanner (Epson Perfection V850 Pro, Japan) at a resolution of 400 dpi. Images were analyzed with WinRHIZO Pro 2019 (Instruments Régent Inc., Canada).

### Histochemical analysis of Zn localization

Histochemical analysis of Zn localization was performed as described by Gao et al. [26]. In brief, the maize roots were washed four times in 10 mM Na_2_-EDTA and in deionized water, and were then immersed in 10 μM Zinpyr-1 (ab145349; Abcam, USA) for 3 h at room temperature in darkness. The roots were washed with deionized water and placed in 0.9% saline. Fluoresce were detected at 490 nm laser excitation, their collection bandwidth was at 530 nm. The images were collected with a Zeiss LSM 980 microscope. At least 12 roots were observed for each treatment, and representative images are shown.

### Free IAA analysis

Root at where LR began to emerge was sampled and pre-treated as previously described [48]. Samples were collected and frozen in liquid nitrogen. A 100 mg (fresh weight) sample was finely ground in liquid nitrogen and the extracted with 1.5 mL of methanol containing ^2^H_2_-IAA (internal standard; CDN Isotopes) and antioxidant at 4 °C for 24 h. IAA was quantified using UPLC-MS/MS consisting of a ACQUITY UPLC I-class system (Waters Corporation, USA) and Q Extractive high-resolution mass spectrometry (Thermo Scientific, USA). Four independent biological replicates were assessed for each treatment.

### Phytohormone treatments

1-NAA or L-kynurenine (L-Kyn) was dissolved in dimethylsulfoxide (DMSO). Inbred line B73 was grown in hydroponic solutions with 20 mM 1-NAA or 20 μM L-Kyn for 7 days under +P+Zn, +P-Zn, -P + Zn or -P-Zn conditions. The hydroponic solutions without 1-NAA or L-Kyn were supplied with 0.1% (v/v) DMSO to ensure similar growth conditions. At least 10 roots per treatment were sampled for RSA observation.

### Determination of Total P and Zn content

Total P and Zn contents were determined as described by Zhang et al. [49]. The weighed samples were digested with HNO_3_ -H_2_O_2_ in a microwave-accelerated reaction system (CEM, Matthews, NC, USA) until the solution became clear. The total P and Zn contents in the digested solutions were determined by inductively coupled plasma optical emission spectroscopy (OPTIMA 3300 DV, Perkin-Elmer, USA). Reference sample ISE885 (Wageningen University, The Netherlands) was used to calibrate the quantification.

## Supplementary Information

**Additional file 1: Supplemental Table 1.** The sequences of the specific primers in the experiment. **Figure S1.** Effects of P and Zn supply on root system architecture (RSA) of maize. (A) Representative images of maize roots as affected by P and Zn supply (images were captured with a flatbed scanner). Scale bars = 1 cm. (B) The effects of P and Zn supply on the total root length of maize. Values are means ± SD (n = 12). Means with the same letter are not significantly different at P < 0.05 according to the LSD test. **Figure S2.** Effects of P and Zn supply on the lengths of primary roots and total roots (primary and lateral roots) of maize. Values are means ± SD (n = 12). Means with the same letter are not significantly different at P < 0.05 according to the LSD test. **Figure S3.** Correlation matrix of biological replicates of transcriptome profiles of maize. The color bar represents the Pearson’s correlation coefficient from 0.7 (blue) to 1 (red). **Figure S4.** Validation of RNA-Seq by RT-qPCR. RT-qPCR quantification was normalized to *ZmActin* expression. Error bars represent the standard error of three biological replicates. Means with the same letter are not significantly different at P < 0.05 according to the LSD test. **Figure S5.** Confocal image of fluorescent Zn signals in maize roots under +P+Zn and -P+Zn. Scale bars = 100 μm. **Figure S6.** Original blots of Fig. 7A. **Figure S7.** Original blots of Fig. 7B. **Figure S8.** Effects of P and Zn supply on root system architecture (RSA) of *ZmMIR167b* transgenic maize. Scale bars = 1 cm.

## Data Availability

All data analyzed during this study are included in the supplementary information files, and genotypic data have been deposited in the Sequence Read Archive to NCBI under BioProject PRJNA857180.
