# Supplementary material for: The auxin signaling pathway contributes to phosphorus-mediated zinc homeostasis in maize
Source: BMC Plant Biol. 2023 Jan 10;23:20. doi: 10.1186/s12870-023-04039-8 (PMC9830811; doi:10.1186/s12870-023-04039-8)
Supplement: Supplementary file 1 — Additional file 1: Supplemental Table 1. The sequences of the specific primers in the experiment. Figure S1. Effects of P and Zn supply on root system architecture (RSA) of maize. (A) Representative images of maize roots as affected by P and Zn supply (images were captured with a flatbed scanner). Scale bars = 1 cm. (B) The effects of P and Zn supply on the total root length of maize. Values are means ± SD (n = 12). Means with the same letter are not significantly different at P < 0.05 according to the LSD test. Figure S2. Effects of P and Zn supply on the lengths of primary roots and total roots (primary and lateral roots) of maize. Values are means ± SD (n = 12). Means with the same letter are not significantly different at P < 0.05 according to the LSD test. Figure S3. Correlation matrix of biological replicates of transcriptome profiles of maize. The color bar represents the Pearson’s correlation coefficient from 0.7 (blue) to 1 (red). Figure S4. Validation of RNA-Seq by RT-qPCR. RT-qPCR quantification was normalized to ZmActin expression. Error bars represent the standard error of three biological replicates. Means with the same letter are not significantly different at P < 0.05 according to the LSD test. Figure S5. Confocal image of fluorescent Zn signals in maize roots under +P+Zn and -P+Zn. Scale bars = 100 μm. Figure S6. Original blots of Fig. 7A. Figure S7. Original blots of Fig. 7B. Figure S8. Effects of P and Zn supply on root system architecture (RSA) of ZmMIR167b transgenic maize. Scale bars = 1 cm. [file 12870_2023_4039_MOESM1_ESM.docx]

**Supplemental Table 1.** The sequences of the specific primers in the experiment

| **Name** | **Sequences (5´-3´)** |
| --- | --- |
| **MIR399-anti** | **CAGGGCAACTCTCCTTTGGCA** |
| **U6 -anti** | **AGGGGCCATGCTAATCTTCTC** |
| **Zm167g-anti** | **CAGATCATGCTGGCAGCTTCA** |
| **Zm00001d032850 real F** | **CTCCAACTTGCTTGGCTTTATCCTC** |
| **Zm00001d032850 real R** | **GACATCTCCTCGAGCGACTTACC** |
| **Zm00001d038437real F** | **TCGTCTCTCAGGTGCTGGA** |
| **Zm00001d038437 real R** | **GAAGAGCTGGTGGAACGTCA** |
| **Zm00001d027700 real F** | **TTGTTCTGTATCGCGCTCGT** |
| **Zm00001d027700 real R** | **TGAAGCCGTAGACGCTCTTG** |


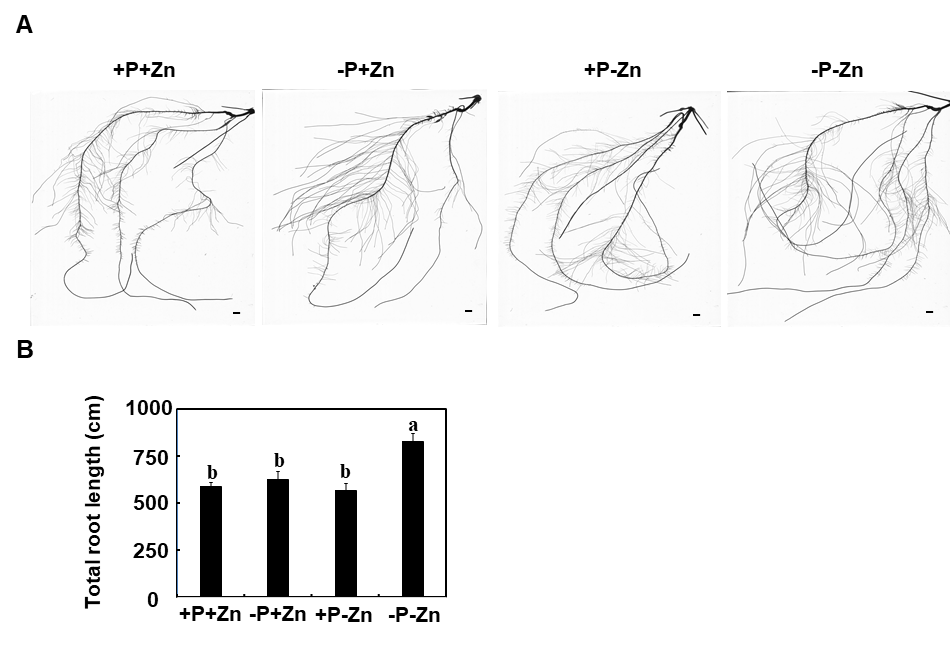


**Figure S1. Effects of P and Zn supply on root system architecture (RSA) of maize.** (A) Representative images of maize roots as affected by P and Zn supply (images were captured with a flatbed scanner). Scale bars = 1 cm. (B) The effects of P and Zn supply on the total root length of maize. Values are means ± SD (n = 12). Means with the same letter are not significantly different at P < 0.05 according to the LSD test.


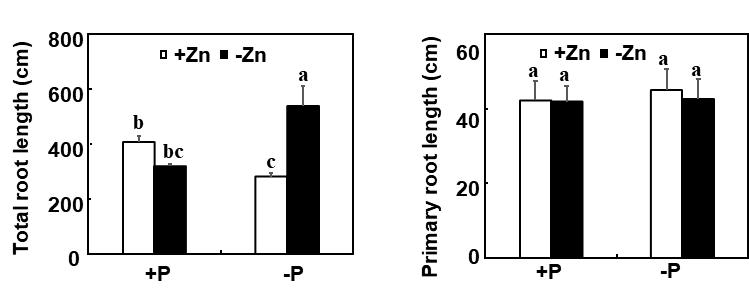


**Figure S2. Effects of P and Zn supply on the lengths of primary roots and total roots (primary and lateral roots) of maize.** Values are means ± SD (n = 12). Means with the same letter are not significantly different at P < 0.05 according to the LSD test.


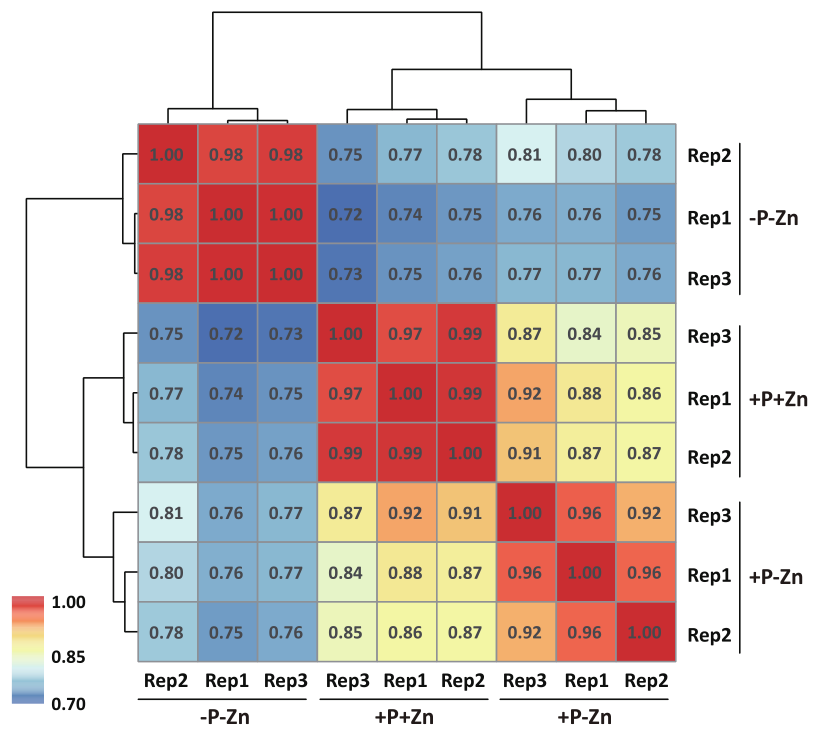


**Figure S3. Correlation matrix of biological replicates of transcriptome profiles of maize.** The color bar represents the Pearson’s correlation coefficient from 0.7 (blue) to 1 (red).


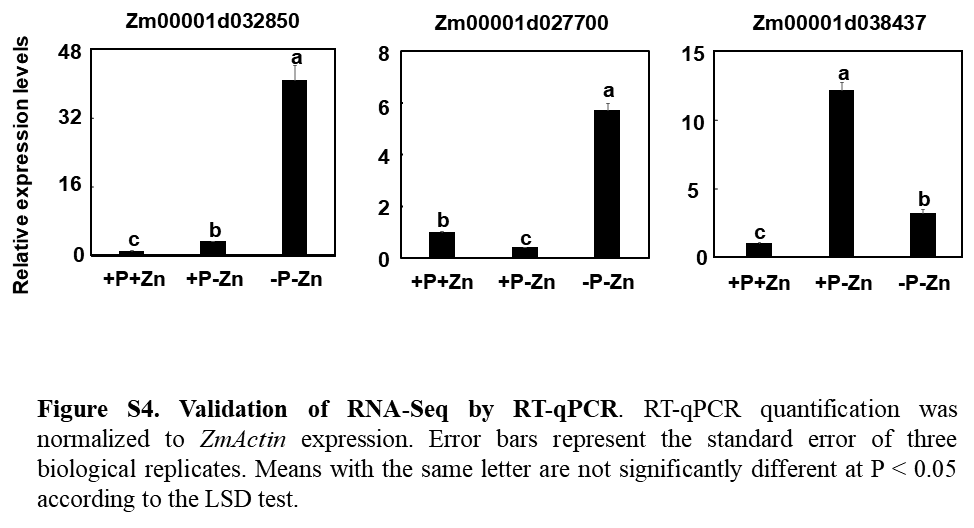


**Figure S4. Validation of RNA-Seq by RT-qPCR**. RT-qPCR quantification was normalized to *ZmActin* expression. Error bars represent the standard error of three biological replicates. Means with the same letter are not significantly different at P < 0.05 according to the LSD test.


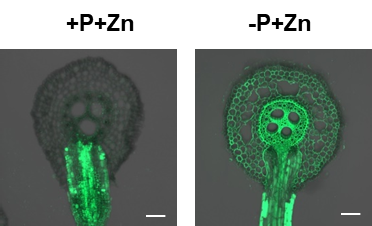


**Figure S5. Confocal image of fluorescent Zn signals in maize roots under +P+Zn and -P+Zn.** Scale bars = 100 µm.


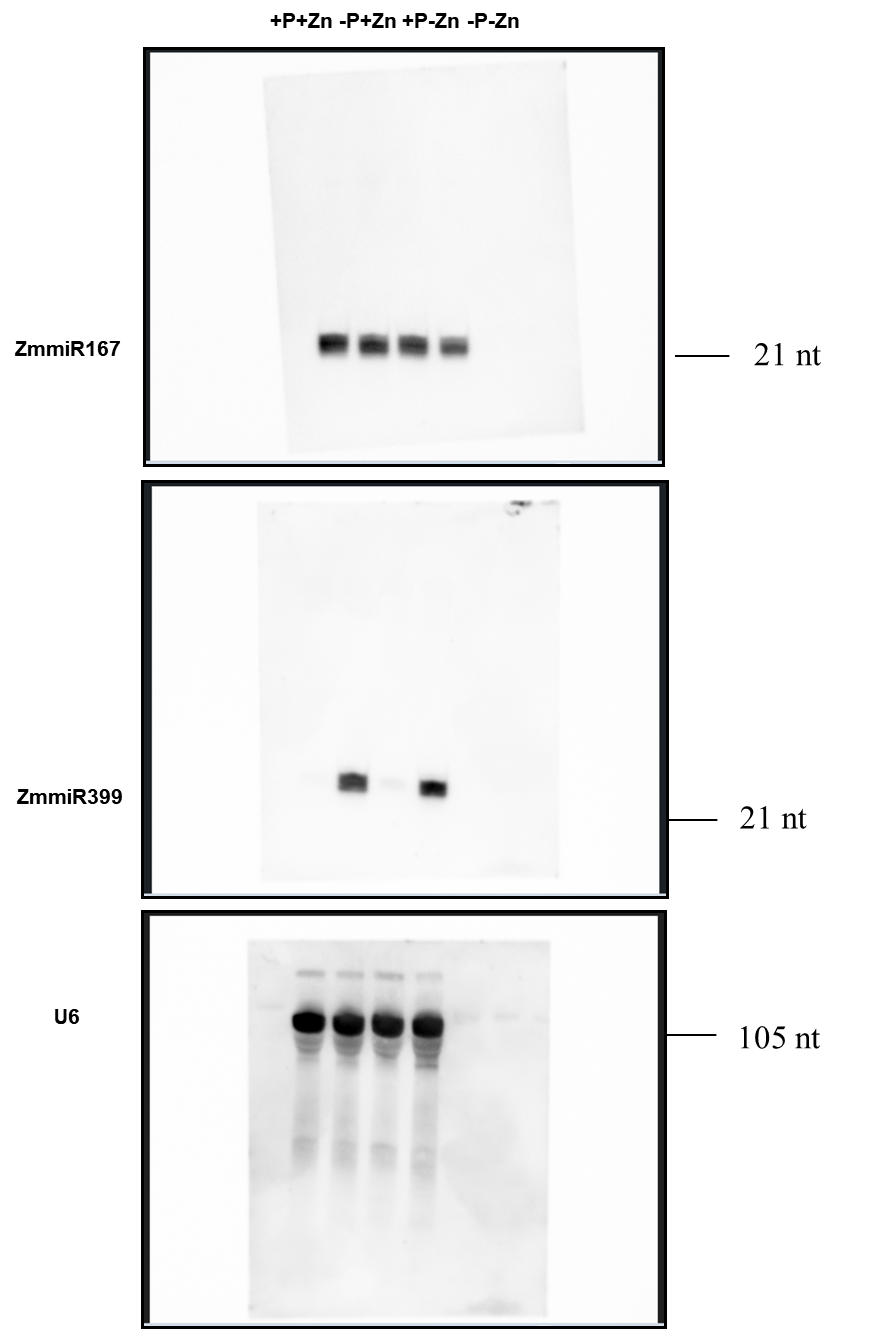


**Figure S6. Original blots of Figure 7A.**


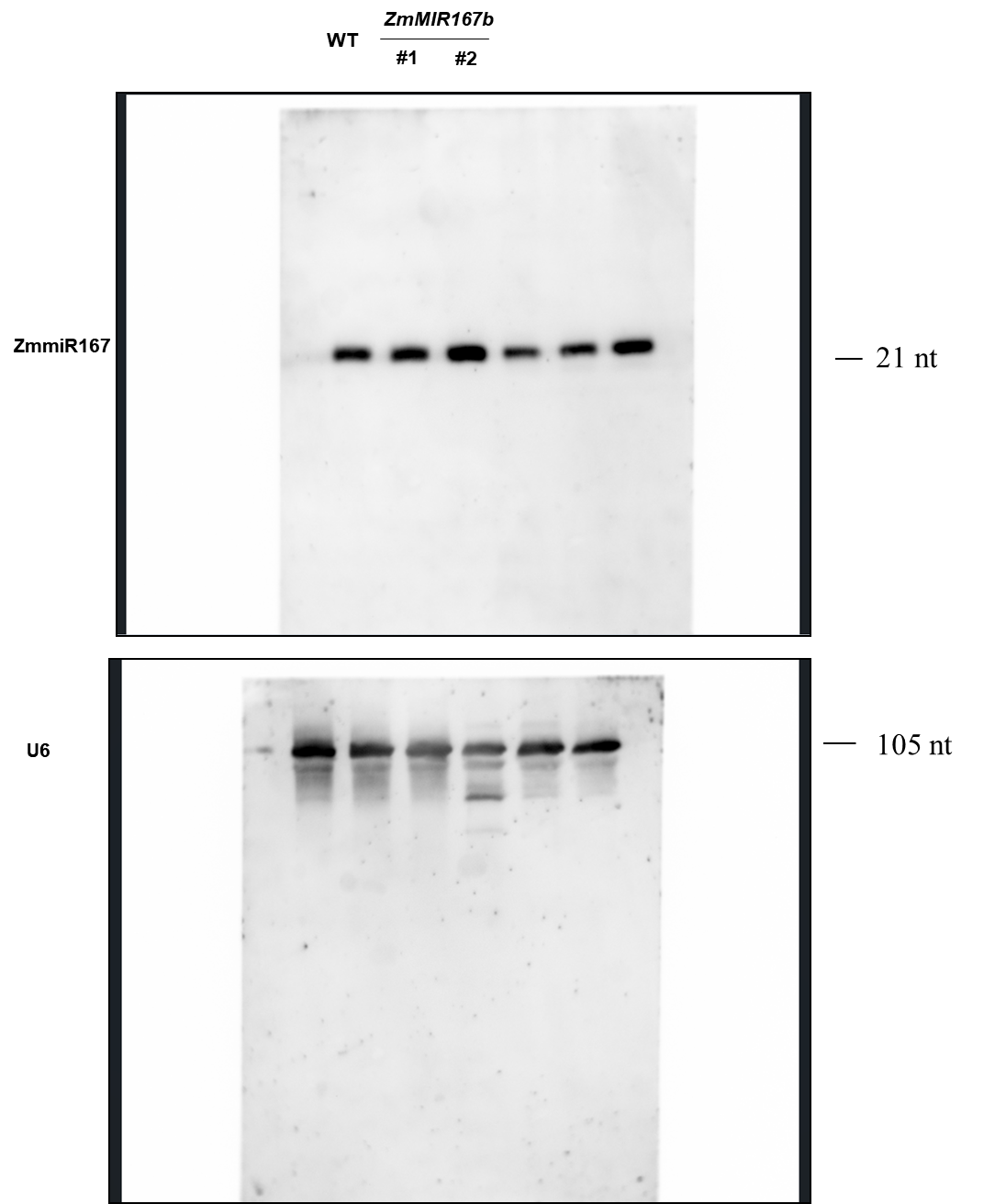


**Figure S7.** Original blots of Figure 7B.


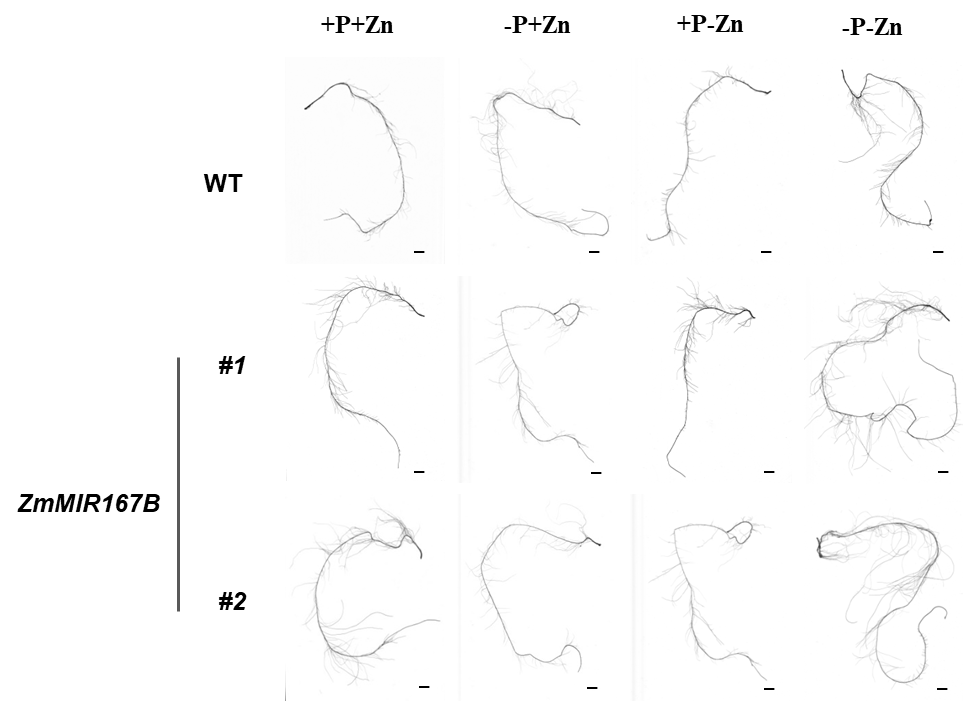


**Figure S8. Effects of P and Zn supply on root system architecture (RSA) of *ZmMIR167b* transgenic maize.** Scale bars = 1 cm.
